# Targeting intrinsically disordered proteins involved in cancer

**DOI:** 10.1007/s00018-019-03347-3

**Published:** 2019-10-30

**Authors:** Patricia Santofimia-Castaño, Bruno Rizzuti, Yi Xia, Olga Abian, Ling Peng, Adrián Velázquez-Campoy, José L. Neira, Juan Iovanna

**Affiliations:** 1 Centre de Recherche en Cancérologie de Marseille (CRCM), INSERM U1068, CNRS, UMR 7258, Aix-Marseille Université and Institut Paoli-Calmettes, Parc Scientifique et Technologique de Luminy, 163 Avenue de Luminy, 13288 Marseille, France; 2 CNR-NANOTEC, Licryl-UOS Cosenza and CEMIF.Cal, Department of Physics, University of Calabria, Via P. Bucci, Cubo 31 C, Arcavacata di Rende, 87036 Cosenza, Italy; 3 Chongqing Key Laboratory of Natural Product Synthesis and Drug Research, School of Pharmaceutical Sciences, Chongqing University, No. 55 Daxuecheng South Road, Chongqing, 401331 People’s Republic of China; 4 Instituto de Biocomputación y Física de Sistemas Complejos (BIFI), Joint Units IQFR-CSIC-BIFI, and GBsC-CSIC-BIFI, Universidad de Zaragoza, 50009 Zaragoza, Spain; 5 Aragon Institute for Health Research (IIS Aragon), Zaragoza, Spain; 6 Centro de Investigación Biomédica en Red en el Área Temática de Enfermedades Hepáticas y Digestivas (CIBERehd), Madrid, Spain; 7 Departamento de Bioquímica y Biología Molecular y Celular, Universidad de Zaragoza, 50009 Zaragoza, Spain; 8 Instituto Aragonés de Ciencias de la Salud (IACS), 50009 Zaragoza, Spain; 9 Aix-Marseille Université, CNRS, Centre Interdisciplinaire de Nanoscience de Marseille, UMR 7325, Equipe Labellisée Ligue Contre le Cancer, Parc Scientifique et Technologique de Luminy, 163 Avenue de Luminy, 13288 Marseille, France; 10 Fundacion ARAID, Government of Aragon, 50018 Zaragoza, Spain; 11 Instituto de Biología Molecular y Celular, Universidad Miguel Hernández, Avda. del Ferrocarril s/n, Elche, 03202 Alicante, Spain

**Keywords:** Cancer, Drug design, Protein function, Intrinsically disordered protein, NUPR1, Stress response, Pancreatic ductal adenocarcinoma, Protein–protein interactions

## Abstract

Intrinsically disordered proteins (IDPs) do not have a well-defined structure under physiological conditions, but they have key roles in cell signaling and regulation, and they are frequently related to the development of diseases, such as cancer and other malignancies. This has converted IDPs in attractive therapeutic targets; however, targeting IDPs is challenging because of their dynamic nature. In the last years, different experimental and computational approaches, as well as the combination of both, have been explored to identify molecules to target either the hot-spots or the allosteric sites of IDPs. In this review, we summarize recent developments in successful targeting of IDPs, all of which are involved in different cancer types. The strategies used to develop and design (or in one particular example, to repurpose) small molecules targeting IDPs are, in a global sense, similar to those used in well-folded proteins: (1) screening of chemically diverse or target-oriented compound libraries; or (2) study of the interfaces involved in recognition of their natural partners, and design of molecular candidates capable of binding to such binding interface. We describe the outcomes of using these approaches in targeting IDPs involved in cancer, in the view to providing insight, to target IDPs in general. In a broad sense, the designed small molecules seem to target the most hydrophobic regions of the IDPs, hampering macromolecule (DNA or protein)–IDP interactions; furthermore, in most of the molecule–IDP complexes described so far, the protein remains disordered.

## Introduction

Intrinsically disordered proteins (IDPs) do not have distinct, well-defined secondary and tertiary structures, because of their remarkable backbone flexibility [1, 2]. Their sequence has a large percentage of charged and polar residues, and a low percentage of hydrophobic, bulky side-chains [2]. IDPs are present in many organisms and their proportion increases from bacteria to higher species [3]. In fact, IDPs involved in gene regulation, protein networks, and cell signaling are over-represented, and therefore, they have been considered during the last decade as potential drug targets [4]. For instance, approximately 70% of human cancer-associated proteins have been predicted to contain relatively long unstructured regions [3, 5]. Unfolded regions in those proteins are involved in protein–protein (PP) or other biomolecular interactions, interacting with different partners in many-to-one and one-to-many binding equilibria (i.e., acting as “hubs”) [6]. Furthermore, they can also bind to their partners in different conformations or cause dissociation (the so-called “moonlighting” [7]). Their highly flexible nature makes them suitable for post-translational modification, and, in fact, they are often involved in phosphorylation pathways [7, 8].

**Fig. 1:**
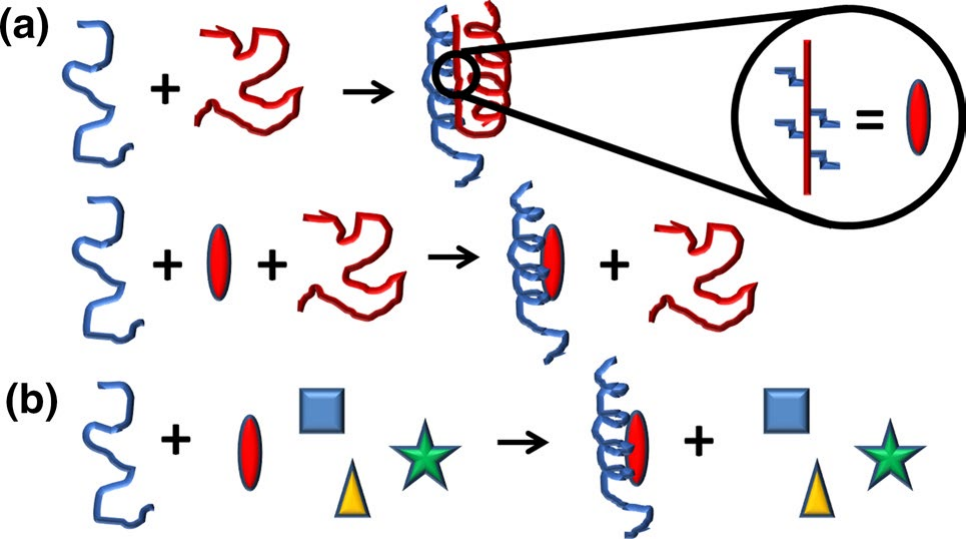
The two approaches used in screening IDPs. The red oval indicates the molecule that binds to the PP interface of the target IDP (in blue), and it is designed based on the structural features of the partner protein, which, in our example, is another IDP (in red), but it could be a well-folded protein (**a**). In the second approach (**b**), a library of different compounds (represented as shapes with different colors) is used to find out whether one of them is captured by the IDP (in our example the red oval again). In both examples, we have assumed that the IDP folds after binding to the molecule, but it could also remain disordered upon binding

During the binding of an IDP to a macromolecule (usually another protein), large interfaces are involved, resulting in very specific, but comparatively weak, interactions [8, 9]: the appropriate partner provides the required complementary surface generating sufficient enthalpy gain to compensate for the loss of entropy occurring upon binding. However, when we design a small molecule, capable of binding the IDP, hampering its protein–protein interactions (PPIs), the understanding of the thermodynamics of the binding reaction is mostly lacking, since the designed compound does not usually have a large interface to interact with. Targeting the PP interface of an IDP with small designed molecules can be envisioned from two general perspectives. First, specific short regions of the intrinsically disordered (ID) sequence, which are known (or predicted) to be involved in binding, are matched by structure-based rationally designed small molecules. These molecules will replace the recognition region of the IDP in the binding to its partners [10]. The second perspective is achieved when a small molecule, identified by a particular screening protocol, binds to a segment of the IDP (usually, but not always, the hot-spot region, as discussed in the sections below); this second approach involves a “fishing” protocol similar to those used for the drug screening on well-folded proteins [11] (Fig. 1). In both approaches, the local nature of the binding interactions with the small molecule reduces the chain entropic costs associated with the folding-upon-binding reaction that takes place usually for most of the IDPs. In addition, the last approach may lead to a competitive small molecule directly blocking the binding region in the IDP (orthosteric binding) or to a non-competitive small molecule binding to another binding site (allosteric binding) in the IDP, trapping the IDP into an inactive conformation by hindering its required functional conformational change. The relative importance of the desolvation entropy contribution (entropic gain favoring the binding originated by the hydrophobic effect and related to the release of water molecules from the binding interface to the bulk solution) and the conformational entropy contribution (entropic loss opposing the binding associated with the change in degrees of freedom of structural elements of the interacting molecules, as a result of the multimolecular assembly along the binding process) is difficult to predict or estimate. The desolvation entropy gain depends on: (1) the amount of hydrophobic groups located in the binding interface; and (2) the net number of water molecules released upon binding. The conformational entropy loss depends on: (1) the degree and extent of flexibility of both interacting molecules; and (2) their structural disorder before and after the binding process. If a disordered protein, interacting with a binding partner, undergoes a global folding, as a result of the interaction leading to a well-defined ordered binding interface, the conformational entropy loss will be larger than that for a disordered protein that only folds locally or even remains globally unstructured upon binding and establishes a fuzzy complex with the binding partner.

In this review, we provide and discuss recent examples of IDPs, involved at different stages in several kinds of cancers, which have been successfully targeted by small molecules. We aim to obtain general conclusions on the current protocols used in targeting IDPs and to foresee future approaches and developments. The mechanisms by which the molecules act seem to be similar in most of the assayed IDPs: that is, hampering PP, or DNA–protein, interactions, mainly taking advantage of hydrophobic effects, although the fine details are protein-dependent (and then, cancer-dependent). Most of the successfully targeted IDPs are implicated in cellular stress mechanisms. Cancer cells, in general, grow under a hypoxic microenvironment, with low contribution of nutrients, high mechanic strain, and various other constraining conditions, which activate stress proteins [12, 13]. Stress protein-dependent mechanisms can help cancer cells to adapt to such harsh environmental conditions. Thus, cancer cells growing under stress conditions become highly dependent on the function of those stress proteins which are mainly ID [14].

## Targeted IDPs involved in cancer

### The c-Myc/Max system: blocking IDP hetero-dimer structure and function

**Fig. 2:**
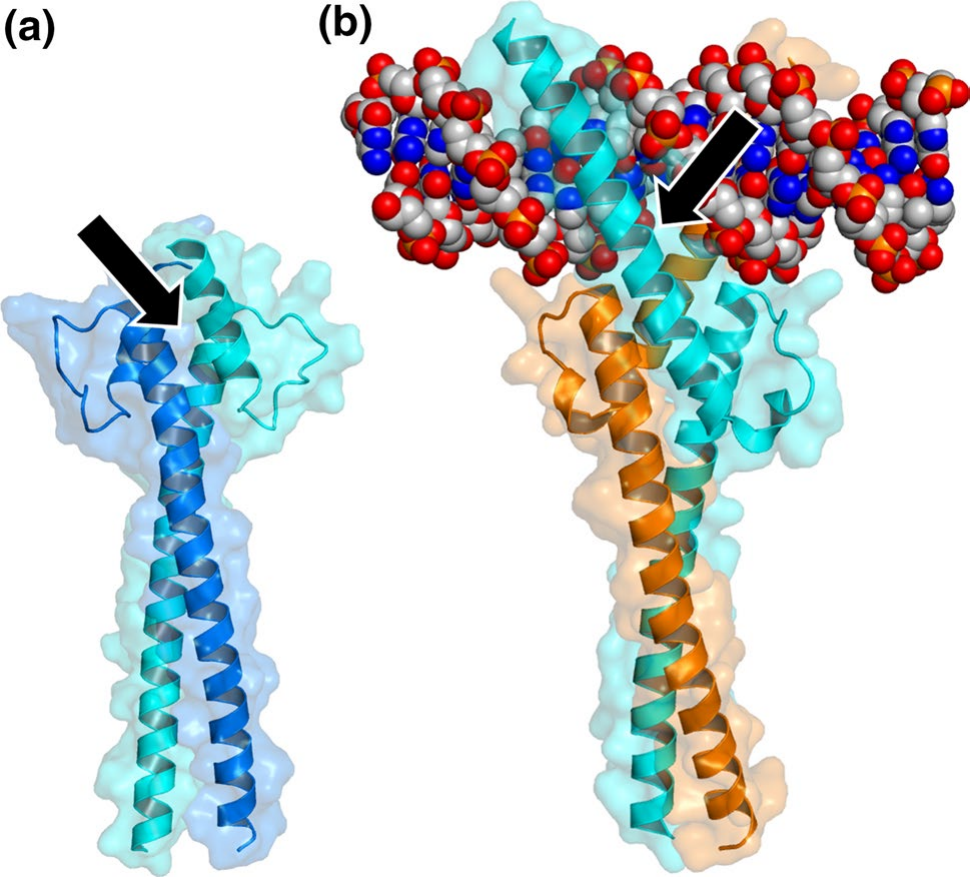
Structure of the homodimer of Max (PDB entry: 5I4Z) [104] (**a**) and the heterodimer with c-Myc and DNA [17] (PDB number: 1NKP) (**b**). The arrow in **a** indicates the homodimer interface; and that in **b** indicates the protein-DNA interface. The figure was produced with PyMOL [105]

The oncogenic transcription factor (TF) c-Myc is involved in cell growth, apoptosis, and metabolic processes [15, 16]. Structurally, it is composed by two independent regions: an N-terminal transactivating domain and a C-terminal helix-loop-helix leucine zipper region (Fig. 2). The c-Myc binds through the latter polypeptide patch to the partner protein Max, which uses a similar domain and is also capable of forming homo-dimers. Both leucine zippers are disordered before binding each other, but, upon binding, both chains become ordered forming a left-handed, four helix-bundle [17]. The resulting hetero-dimer (which is the functional species) binds DNA, and modulates gene expression, transcriptional activation, and apoptosis [16, 18]. Deregulation of c-Myc is mostly associated with aggressive tumors in breast, lung, cervix, and hematopoietic organs [19], as well as with deregulation of genes controlled by RNA polymerases [20].

This system was the first example, showing that an IDP could be targeted with small molecules or peptides. There are two possible approaches to accomplish the targeting: first, designing small molecules that impede the hetero-dimerization; second, identifying molecules or peptides hampering the binding of the formed hetero-dimer to DNA (but not the hetero-dimer formation). There have been two attempts of using the former approach and one using the latter, which are described in the next paragraphs.

**Fig. 3:**
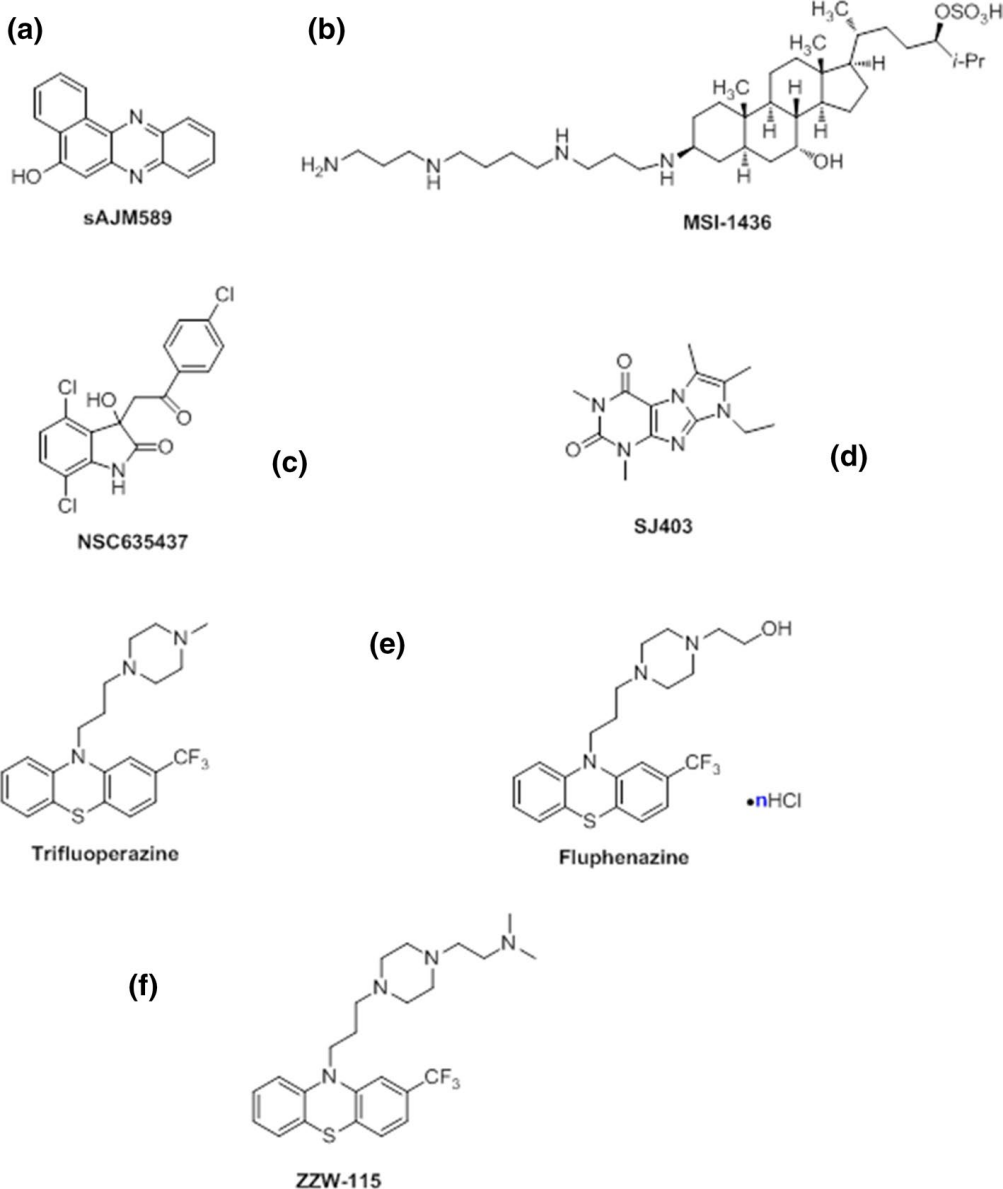
Structure of compounds used in targeting IDPs: **a** sAJM589 for the c-Myc system; **b** MSI-1436 for C-PTIB; **c** NSC635437 for the EW-FLI1 fusion protein; **d** the compound targeting p27-KID; **e** TFP (left side) and Fluphenazine hydrochloride (right side) identified for NUPR1; **f** the ZZW-115 compound obtained by ligand-based design

Inhibitors of c-Myc/Max association have been first found using high-throughput screening of small molecules or peptide-mimetic libraries, based on the application of fluorescence resonance energy transfer (FRET) of fluorescent derivatives of c-Myc and Max [21, 22] together with two-yeast hybrid (TYH) techniques [23]. Then, at a later stage, other biophysical techniques such as circular dichroism (CD) and NMR have been used to assess the binding in vitro [20, 24]. Several compounds have been found to bind simultaneously and independently to different hydrophobic regions of the c-Myc monomer [25] shifting the on-going equilibria among its disordered conformations. The molecule blocks a particular conformation of c-Myc, and this conformation is incapable of interacting with Max [24, 26, 27], and hence, it cannot carry out its related biological functions. That is, there is a shift in the population of conformers populated by the IDP, and therefore, the heterodimer formation is not hampered by direct interaction of the compound with the hetero-dimeric interface (indicated by an arrow in Fig. 2a) but rather by an increase in the population of the monomer, which has a non-competent conformation to form the dimer. However, although the binding of the small molecules induces local structural changes in the protein, mainly around hydrophobic patches, it leaves the rest of the polypeptide chain disordered (that is, the overall disorder through the protein chain is maintained). These local structures are different to the well-folded bundle spanned by the protein when it is bound to Max. IC_50_ of sAJM589, the best-discovered compound, was 1 µM [28] (Fig. 3a).

In the second attempt, fluorescence polarization screenings of c-Myc have allowed the identification of two compounds, which prevent DNA binding and transcription of the heterodimer. Therefore, in this case, the function of the hetero-complex, and not its formation, is directly abolished. However, the molecules lack specificity, as they block the DNA binding of Max homodimer as well [29]; although there are not structural details reported, binding of the compound should occur at the protein–DNA interface (Fig. 2b).

The third attempt of targeting c-Myc also uses the second approach described above (Fig. 1). A variant of the helix–loop–helix domain of c-Myc was designed, containing four mutations in the leucine zipper region. Therefore, the molecule is a variant of the wild-type peptide. This mutant (achieved through chromosomal insertion of a doxycycline-inducible gene) was capable of forming dimers with c-Myc and Max proteins, competing with all the heterodimers of the c-Myc family for their target promoter; therefore, the peptide hampers the function of the dimer, and it could be considered a transgenic tool to inhibit c-Myc in vitro [30, 31]. Although there are not structural details reported, it is tempting to suggest that the heterodimers formed by the mutant peptide do not have the same conformation as the wild-type dimer in the protein–DNA interface (Fig. 2b). Recently, it has been shown that this peptide is capable of penetrating the cells, reverting the expression of c-Myc-related gene signatures, and shifting equilibria where c-Myc is involved throughout the genome [32]. Taken together, the results suggest that the peptide is very specific and the basis of its function is the DNA binding.

To summarize, the disordered protein c-Myc can be targeted by small molecules, that bind to hydrophobic regions of the isolated polypeptide, leaving its structure mainly disordered. These molecules are capable of hampering hetero-dimer formation.

### The disordered C-terminal region of PTP1B (C-PTP1B): allosterism in regulation of the function of an ID region

The protein tyrosine phosphatase 1B (PT1B) is over-expressed in breast tumors together with HER2 [33]. Its disordered non-catalytic C-terminal region (C-PT1B) has a regulatory function [34]. The use of a natural product (trodusquemine, MSI-1436) inhibits the metabolic function of PT1B, by binding to its catalytic site [35]. However, MSI-1436 (Fig. 3b) is also a non-competitive inhibitor of PTP1B, which binds to C-PTP1B, inducing conformational changes [36]. Although C-PTP1B is mainly disordered, it has a propensity to populate helical conformations around two distinct regions. In fact, several biophysical techniques [FRET, small angle X-ray scattering (SAXS), size exclusion chromatography (SEC), and state-of-the-art NMR] have been used to elucidate the flickering helical structure in C-PTP1B and how it changes in the presence of MSI-1436. The compound binds to residues of the second, flickering helical region of C-PTP1B (which is slightly more hydrophobic than the other helical patch), and to another additional site close to the catalytic domain. Binding to C-PTP1B induces allosteric changes in the protein, locking the whole protein in an inactive state. At this stage, although C-PTP1B in complex with the compound has a more compact hydrodynamic radius than that of the isolated protein [36], it is not known whether the complex formed has a fuzzy conformation, remaining disordered as it happens with the c-Myc/Max complex and the assayed molecules. In addition, MSI-1436 disrupts HER2 signaling and inhibits tumorigenesis in xenografted mice, showing that PTP1B is, as well, a valid target to treat breast cancer [36].

Therefore, in this example, the targeting of the disordered protein with small molecules does not hamper directly the interaction with other proteins (as in the case of c-Myc/Max system), but rather induces allosteric conformational changes, which impedes subsequent binding (and its biological function).

### The EWS–FLI1 fusion protein: designing small compounds or peptides to hamper PPIs of an IDP

There are some leukemia and sarcomas that carry non-random chromosomal translocations, encoding novel fusion TFs. These TFs are produced under cellular stress conditions, and are essential to initiate and maintain the molecular pathogenesis of the cancer. Although the majority of these fusion proteins are TFs, some of them are also involved in phosphorylation processes. The genomic fusion of TET family members, such as Ewing’s sarcoma oncoprotein (EWS), usually involves an *ets* gene (erythroblastosis virus E26 transforming sequence gene) such as the *FLI1* [37]. This fusion yields the Ewing’s family of oncogenic proteins (EWS-fusion proteins or EFPs). The N-terminal region of the EFP belongs to the EWS, which contains the transcriptional activation domain (TA); and the C-terminal region comes from the fusion partner, containing the DNA-binding promoter specificity, which determines tumor phenotype. The function of the TA is conferred by the presence of multiple tyrosines at different polypeptide sites.

Biophysical and computational studies have shown that EWS–FLI1 is an IDP [38, 39]. The binding of the fusion EWS–FLI1 protein to RNA helicase is essential in tumor maintenance in Ewing’s sarcoma family tumors [37]; the binding region of the RNA helicase involves residues 647–1075, and that polypeptide patch is not used in binding to other biomolecules. A compound screening using surface plasmon resonance (SPR) allowed the identification of a compound, NSC635437 (Fig. 3c), which binds to EWS-FLI1 [40]. No clues have been provided about the possible local structure acquired by EFP upon binding to the molecule, but computational studies suggest that binding to any compound leaves EWS–FLI1 disordered [37], forming fuzzy complexes (as with the c-Myc/Max example). Although structural details of the EFP binding region to the drug are lacking, as well as any other structural details on other protein regions, it is tempting to suggest (based on mutational studies on natural partners [37]) that some tyrosines along the sequence are involved in the binding (and hydrophobic interactions). An improved molecule developed starting from NSC635437 has led to [40]: (1) disruption of the interaction between the RNA helicase and EWS–FLI1; (2) apoptosis of cancerous cells; and (3) decrease of tumor volume in Ewing’s sarcoma xenografted mice.

The same research group has also found a peptide [40], comprising residues 823–832 from RNA helicase that is capable of blocking the binding between EWS–FLI1 and the intact RNA helicase, suggesting that NSC635437 and the peptide bind to the same fusion protein site (although allosteric processes cannot be fully excluded). However, no clues have been provided whether the bound RNA-helicase-derived peptide leaves EWS–FLI1 disordered (that is, whether a fuzzy complex is formed). Therefore, the authors have used both approaches outlined above (either design of a peptide mimicking the binding region of one of the proteins or screening of a compound library, Fig. 1) to inhibit PPIs between the fusion protein and the helicase.

Therefore, in this example, hydrophobic interactions (involving Tyr residues) are also important in the design of the peptide or the molecules hampering the PPIs of the fusion protein.

### The AF4–AF9 protein system: another complex formed in fusion proteins

Translocations involving the Mixed Lineage Leukemia (*MLL*) gene are involved in leukemogenesis characterized by poor prognosis [41]: fusion of TFs to MLL products is thought to be one of the triggering mechanisms of leukemia [42]. Among the several MLL fusion partners, AF4 and AF9 proteins are quite common. AF9 is a component of biochemically isolated complexes with functions in transcriptional elongation such as the AEP complex, which, in turn, contains the AF4 protein [43]. Both proteins form complexes, either when belonging to the chimeric MLL protein or in their native isolated states [44]. In fact, disruption of the AF4–AF9 complex results in necrotic cell death in cell lines harboring MLL translocations [45], which indicates the importance of the complex formation and its use as possible pharmaceutical target against leukemia.

**Fig. 4:**
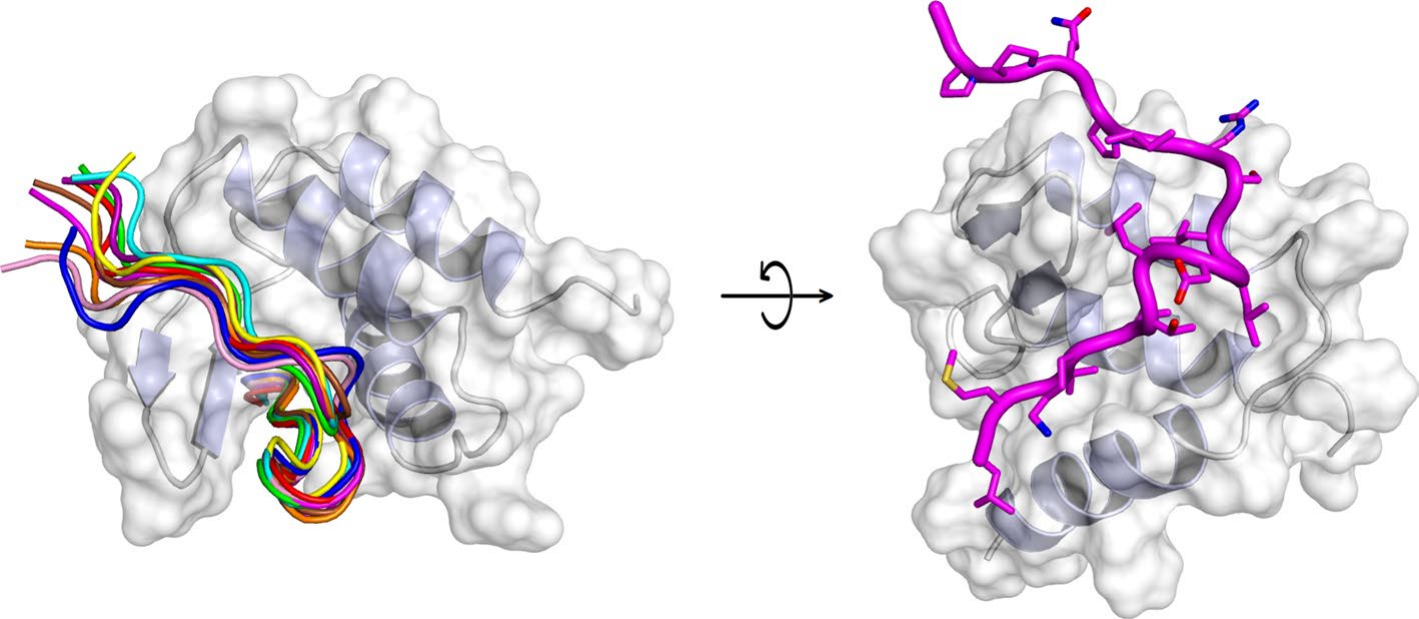
Structure of the complex between the AF9 and the peptide-derived AF4 polypeptide. Ensemble of structures of the bound peptide (left), and details of a single peptide structure in a rotated view of the protein (right). The figure was produced with PyMOL [105] from the PDB entry 2LM0 [47]

AF9 and AF4 are IDPs when isolated in solution and the interacting region of AF4 has been previously identified using TYH techniques [46]. When AF9 binds to a peptide containing the recognition region of AF4 (comprising residues 760–773), it folds (acquiring a conformation with three α-helices and a two-stranded β-sheet, packed on one of the helices), although it maintains a high flexibility [47]. The AF4-derived peptide also folds acquiring several turns of α-helix (Fig. 4); therefore, in this system, the binding of both proteins induces their folding. The interface between both polypeptides is hydrophobic, with several residues of the peptide buried in the hydrophobic core of AF9. This AF4-derived peptide is capable of disrupting the AF4–AF9 complex both in vitro and in vivo, inhibiting the proliferation of leukemia cells (via apoptosis) with chromosomal translocation expressing MLL–AF4 fusion genes [44]. In addition, the peptide did not alter the proliferative capacity of hematopoietic progenitor cells.

Therefore, in this example, knowledge of the PPI interfaces of both proteins—corresponding to the application of the first approach described above—allows the identification of an inhibitor, as it occurs with the c-Myc/Max one. However, conversely to that system, the inhibitor-peptide/AF9 complex yields two well-folded polypeptide chains, although AF9 keeps a high flexibility. Furthermore, the regions involved in binding in both polypeptide chains have a large number of implicated hydrophobic residues [as it happens in the c-Myc/Max system and in the EWS–FLI1 one].

### The p27 system: drug targeting of an IDP using screening of fragment libraries

p27Kip1 (or p27, kinase inhibitory protein 1, and also known as CDKN1B) is a modulator of cyclic-dependent kinases (cdk), that controls eukaryotic cell division [48, 49]. The N terminus of the protein contains the kinase inhibitory domain of p27 (p27-KID, residues 28–90 in p27); this domain binds to, and therefore, modulates (by phosphorylation of its tyrosines) the activity of Cdk2/cyclin complexes. This regulation is incorrectly activated in chronic myelogenous leukemia and breast cancer [50, 51]. In addition, phosphorylation of this domain at a threonine is related to increased metastatic processes and cell migration [52, 53]. The p27-KID is an IDP [54, 55].

It has been hypothesized that, if a small molecule could bind to p27-KID, this could induce a conformational change in the disordered polypeptide chain, which would yield an incompetent species incapable of binding to the Cdk2/cyclin complexes (in a similar reasoning to that used for the c-Myc/Max system) [55]. Using NMR for screening a fragment compound library, nine molecules are capable of blocking the p27-KIP/Cdk2/cyclin complex formation, with their interactions with the IDP being described at atomic level. These compounds could be classified in two groups, each of which binds to different regions of p27-KID. The region of p27-KID involved in the binding has a large amount of hydrophobic residues [as it occurs in the EWS–FLI1 system; the AF4–AF9 one; and the c-Myc/Max complex]. The binding of the small-molecular inhibitors hampers the ability of p27 to sequester the Cdk2/cyclin complex, restoring partially the Cdk2/cyclin activity. Although p27-KID remains disordered upon binding to the small molecules, the binding induces a shift in the conformational equilibria of the IDP [54], as judged by NMR relaxation measurements of the complex with one of the compounds, again in analogy with the behavior of the c-Myc/Max system.

It must be pinpointed that for this system, a peptide resembling the Cdk2/cyclin interface involved in binding to p27-KID has not been used as an inhibitor of the interactions between the two proteins, but a small organic inhibitor. We think that it should be interesting to explore the possibility of finding a peptide capable of binding the same PPI interface as the screened fragments, which could induce folding of the p27-KIP, and mimicking the polypeptide region of the Cdk2/cyclin complex.

To sum up, in this case, a library was screened to identify the compounds targeting the PPI interface of p27-KID (as it happens with c-Myc/Max system). The molecules bind to the hydrophobic regions of the protein (as other examples described before). In addition, the protein does not acquire a folded conformation upon binding to any of the small molecules (as it has been described in other systems).

### The p53 system: a master regulator of cell functions with ID regions

The protein p53 is a TF and a master regulator of cellular function, modulating the expression of genes involved in senescence, cell cycle arrest, and apoptosis [56, 57]. The inactivation of p53 results from the overexpression of proteins such as E3 ubiquitin ligases that can down-regulate the function of wild-type p53 through its degradation [58]. Indeed, in un-stressed cells, p53 levels are low due to this rapid ubiquitination and degradation. One of such E3 ligases is MDM2 (murine double minute 2). MDM2 also promotes its own degradation [59]: under cellular stress, MDM2 phosphorylates at specific locations and it is degraded, triggering p53 stabilization [60]. In addition, under similar stress conditions, MDM2 binds p53 and prevents its interaction with the general transcription machinery. The complex between MDM2 and p53 is largely formed by the interaction between the N-terminal domain of MDM2 (residues 1–124) and the N-terminal TA of p53 (residues 1–61, p53-TA). There is a competition for binding to p53-TA, among the N terminus of MDM2 and other co-activators involved in up-regulation of transcription [61]. Under stress conditions, p53-TA undergoes extensive phosphorylation—whose extent depends on the stress situation—hampering the binding to MDM2, and enhancing the binding to other co-activators.

**Fig. 5:**
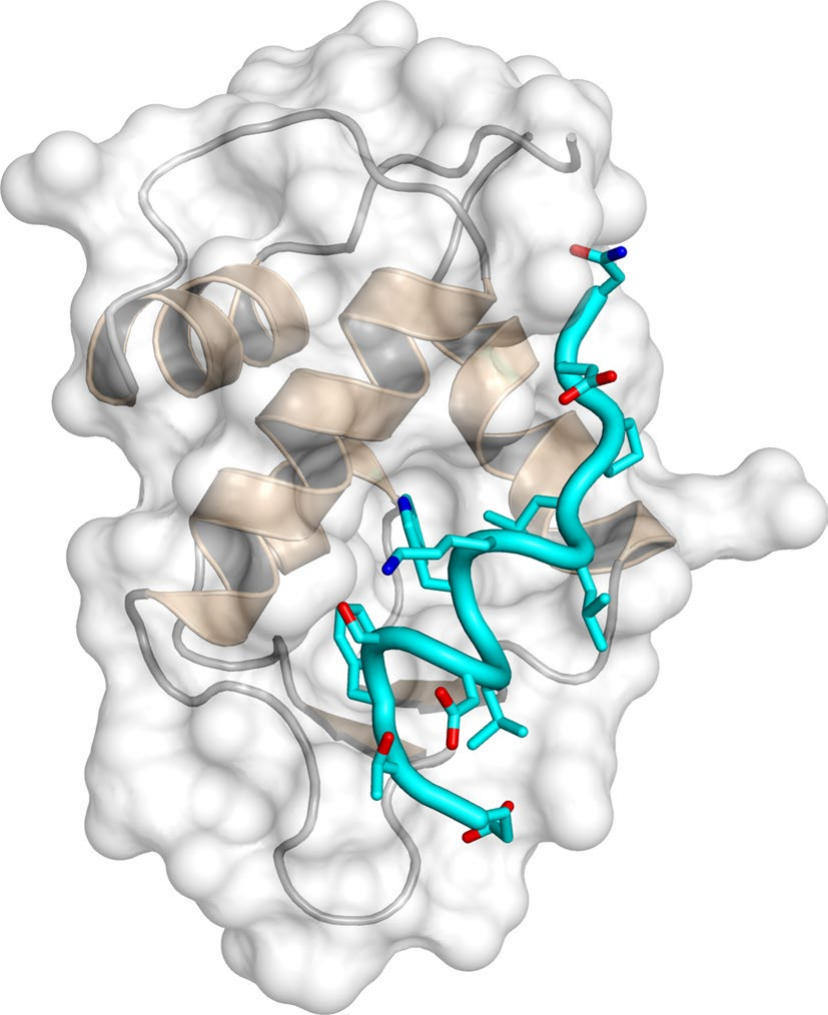
Structure of the complex between the MDM2 and p53-derived peptide from TA region. The figure was produced with PyMOL [105] from the PDB entry 1YCR [68]

In the absence of MDM2, the isolated p53-TA is disordered, with a hydrodynamic radius similar to that of chemically unfolded proteins of the same size, but forming flickering, local elements of secondary structure as judged by the use of several biophysical techniques [62–65]; MDM2 also undergoes a compaction upon binding to p53 [66], but it is well folded in isolation. There are several NMR and crystals structures of the N-terminal domain of MDM2 in complex with peptides derived from p53 or with other molecules [67–69]. In all of those structures (the first one reported in 1996 describes the binding between the N terminal region of MDM2 and a p53-TA-derived peptide [68]), the p53-TA peptide adopts an α-helical conformation. In that folding topology, hydrophobic residues Phe19, Trp23, and Leu26 of the p53-TA (Fig. 5), which are part of the amphipathic helix, are inserted into a hydrophobic pocket on the MDM2 surface. This structure has been used as a starting point to develop inhibitory compounds applying the two approaches used for targeting IDPs: (1) the design of a peptide or peptide mimetic with the features of the p53-TA region; and (2) screening of libraries of compounds that could fit into the hydrophobic groove of MDM2. Both approaches have allowed the screening, design, synthesis and testing (using fluorescence, CD, NMR, and SPR) of several dozens of small inhibitors of this PPI interface [70–72], and even the design of peptides and peptide mimetics which have an affinity similar to that of both intact protein partners [73].

Then, to sum up, this example is a case of folding-upon-binding of a particular ID polypeptide patch (similarly to what happens in the AF4–AF9 system), and, again, hydrophobic residues intervene in the recognition region.

### The NUPR1 system: an essential protein for cancer development

NUPR1 was first described as being activated during the acute phase of pancreatitis [74]. Afterwards, the inducible expression of NUPR1 was discovered to occur under stress conditions caused by many stimuli, in most cell types, and therefore, NUPR1 can be considered a stress-associated protein [75, 76]. Then, NUPR1 was found to be over-expressed in many cancer tissues. At the cellular level, NUPR1 was involved in cancer-associated processes including cell-cycle regulation, apoptosis [77, 78], senescence [79], cell migration and invasion [80], and metastases [81]. Indeed, NUPR1 has attracted attention due to its role in promoting cancer development and progression in the pancreas [82, 83], as NUPR1-dependent effects also mediate resistance to anticancer drugs [84–86]. Remarkably, we have showed that genetic inactivation of NUPR1 antagonizes the growth of pancreatic adenocarcinoma (PDAC) [80, 87], and other laboratories have shown that genetic inactivation of NUPR1 stops the growth of hepatocarcinoma [88], non-small lung cancer [89], cholangiocarcinoma [90], glioblastoma [91], multiple myeloma [92, 93], and osteosarcoma [94]. These findings indicate that NUPR1 is a hub protein involved in several key signaling routes, and it could be considered a promising therapeutic target for the development of new anti-cancer therapies.

Structurally, NUPR1 is an 82-residue-long, monomeric, basic IDP [95–99]. It has two hot spot regions (identified by in silico procedures and protein engineering studies [98]) involved in binding to its natural partners (DNA, prothymosin α, male-specific lethal protein, and the C-terminal domain of RING1B): (1) the 30′s region, where the two tyrosine residues of the protein are located; and (2) the region around Thr68. Both regions are among the most hydrophobic polypeptide patches in the chain [96, 98, 99]. However, in all the complexes, NUPR1 remains disordered.

**Fig. 6:**
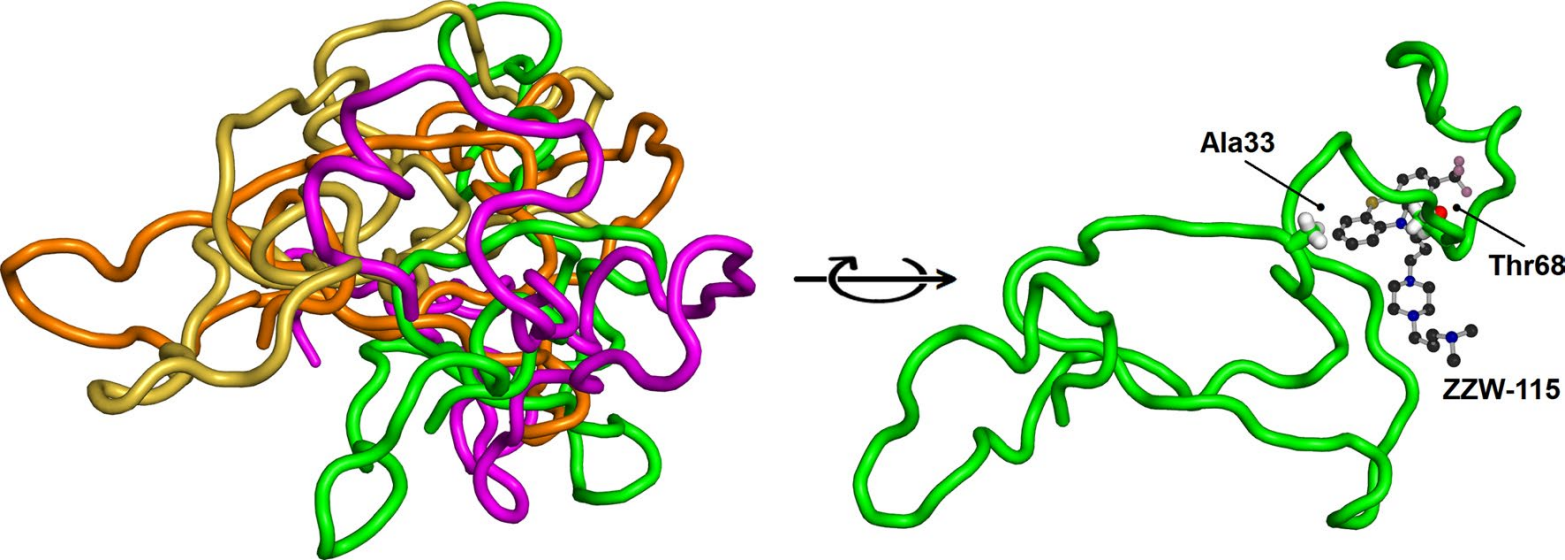
Simulated structures of NUPR1 in the first encounter with the compound ZZW-115. Examples of conformations in the solution ensemble of NUPR1 (left), and details of the binding pocket for ZZW-115 (right). The figure was produced with PyMOL [105] from simulation models previously obtained [97, 100]

We have developed a combination of biophysical, biochemical, bioinformatic [molecular dynamics (MD) simulations], and biological approaches for a molecular screening in vitro, in vivo, in silico, and in cellulo to select potential drug candidates against NUPR1 [100]. In the first step, we have repurposed a well-known drug; and in a second step, we have: (1) improved its efficacy and efficiency; and (2) decreased its side effects. In our approach, we have used the second strategy employed in drug-targeting IDPs (Fig. 1): screening of a commercial library (Prestwick Chemical Library, http://www.prestwickchemical.com/libraries-screening-lib-pcl.html) to identify a lead compound. The screening has been carried out with fluorescence-thermal denaturation. We have selected those compounds with larger variations either in the thermal denaturation midpoints or in the denaturation profiles, and next, we have measured their affinity for NUPR1 using isothermal titration calorimetry (ITC). We have chosen those with the most favourable affinities, with values similar to those found for the NUPR1 natural [78, 96, 98] or non-natural [99] binding partners. We then have combined SAR (structure–activity relationships) by NMR results and MD simulations, carried out independently of each other, to verify that a similar set of residues were affected by the binding of the compound. The most promising compounds identified have been trifluoperazine (TFP) and its structurally related fluphenazine hydrochloride (Fig. 3e). Both compounds bind to both hot spots of NUPR1, which remains disordered upon binding, adopting particular local conformations (as it happened in the c-Myc/Max system) (Fig. 6). Cell viability assays with TFP have led to an IC_50_ ~ 10 µM. Most importantly, tests of TFP in vivo, with human pancreatic cancer cell-derived xenografts implanted into immune-compromised mice, have shown an arrest of the tumor growth in a dose-dependent manner [100]. Unfortunately, high doses of TFP, as that used for PDAC treatment, have also led to neurological side effects, such as strong lethargy and hunched posture, which preclude TFP use in clinics to treat cancers.

In a second step, we have used a rational, in silico ligand-based design relying on a combination of MD and docking, which guided the organic synthesis of TFP-derived compounds. As expected from the in silico studies, the synthesized compounds have shown a stronger affinity in vitro for NUPR1, as indicated by CD, fluorescence, ITC and NMR. The best compound (that with the largest affinity for NUPR1, and within the same order than that of NUPR1 for its natural partners), ZZW-115 (Fig. 5f), kills different kinds of cancer cells with IC_50_ values ranging from 0.84 to 4.93 μM [101]. Most importantly, ZZW-115 shows a dose-dependent tumor regression in xenografted mice, almost leading to tumor disappearance after 30 days of treatment with 5 mg/(kg day), with no apparent neurological effects. At the cellular level, ZZW-115 induces cell death by both necroptotic [as measured by lactate dehydrogenase (LDH) release] and apoptotic (as measured by caspase 3/7 activity) mechanisms, with a concomitant mitochondrial metabolism failure that triggers lower production of ATP and reactive oxygen species (ROS) overproduction [101]. Importantly, these molecular mechanisms are similar to those observed in NUPR1-deficient cells [102] and can be inhibited by Necrostatin-1 (an inhibitor of necroptosis) and Z-VAD-FMK (an inhibitor of caspases). We have also proved that ZZW-115 still exerts its effect by binding to NUPR1, although we could not prove that NUPR1 is the sole protein targeted. In fact, the antitumor effect of ZZW-115 is not influenced by the resistance to others drugs in PDAC treatment, and this compound might modulate another intracellular pathways, by binding also to other macromolecules.

Since NUPR1 is over-expressed in several tumors, we have also evaluated the effect of treating cellular lines derived from those tumors with ZZW-115. Cells such as U87 (glioblastoma), A375 and B16 (melanoma), U2OS and SaOS-2 (osteosarcoma), HT29, SK-CO-1 and LS174T (colon cancer), H1299 and H358 (lung cancer), HepG2 (hepatocarcinoma), PC-3 (prostate), THP-1 (acute monocytic leukemia), Daudi (lymphoma), Jurkat (acute T-cell leukemia), and MDA-MB-231 (breast cancer) have been treated with ZZW-115. The compound is efficient in killing those cells with IC_50_ values ranging from 0.42 to 7.75 μM [101]. These data indicate that ZZW-115 could be an anticancer molecule active in several tissues.

Therefore, ZZW-115 constitutes a promising drug candidate for PDAC with a new molecular mechanism, since it combines the concomitant induction of necroptosis and apoptosis. The compound, obtained by rational ligand-based design, binds to the hydrophobic regions of NUPR1 as in other IDPs and forms a fuzzy complex with the protein.

## Conclusions

Our work and examples from other laboratories indicate that targeting of IDPs is feasible (even in proteins not involved in cancer development [103]). In all cases described so far, the molecules work by hampering the DNA–protein or protein–protein interactions in which the IDP is implicated. The binding of the designed compounds in IDPs mainly involves, as it happens with well-folded proteins, protein hydrophobic regions. Interestingly enough, in most of the targeted IDPs, binding of the molecules does not induce a folding of the polypeptide chain of the disordered protein; in fact, protein-folding-upon-binding only occurs when the molecule is a peptide [as in the AF4–AF9 system or in the MDM2–p53 complex]. It should be interesting to elucidate whether this is a particular feature of the corresponding IDP, or alternatively it is due to the fact that the smaller size of a molecule (compared to that of a peptide) hampers the protein in acquiring a well-folded conformation, but influences enough to shift the conformational equilibrium of the protein and then obstructing its function. Thus, folding-upon-binding might require the cooperative target of several, appropriately distributed interaction hot spots.

The fact that either screening of libraries or dissection and study of PPIs in the formed complexes of IDPs with other biomolecules can be used in targeting IDPs indicates that the drug-design techniques employed in well-folded proteins can be used in IDPs, with slight variations. Moreover, the designed compounds against IDPs encompass, as in well-folded proteins, either small, synthetic organic molecules, or peptides (and their derivatives). Differences among the examples described rely on how molecular design is carried out against IDPs, since this design is essentially ligand-based, as opposed to structure-based in well-folded proteins. This is due to the fact that there is not a single structure for the corresponding IDP. However, the design of drugs targeting IDPs is still in its infancy, and additional examples are expected to shed light on details in common among successful attainments, as well as novel ways for pursuing further rational drug design strategies.

## Abbreviations

CD: Circular dichroism
cdk: Cyclin-dependent kinase
C-PTP1B: Disordered C-terminal region of protein tyrosine phosphatase 1B
EFP: Ewing’s fusion protein
EWS: Ewing’s sarcoma oncoprotein
FRET: Fluorescence resonance energy transfer
HER2: Human epidermal growth factor receptor 2
ID: Intrinsically disordered
IDP: Intrinsically disordered protein
ITC: Isothermal titration calorimetry
KID: Kinase inhibitory domain
LDH: Lactate dehydrogenase
MD: Molecular dynamics
MDM2: Murine double minute 2
MLL: Mixed lineage leukemia
NMR: Nuclear magnetic resonance
NUPR1: Nuclear protein 1
p53-TA: The TA of p53, comprising residues 1–61 of the whole intact p53 protein
PDAC: Pancreatic ductal adenocarcinoma
PP: Protein–protein
PPI: Protein–protein interaction
PTP1B: Protein tyrosine phosphatase 1B
ROS: Reactive oxygen species
SAXS: Small-angle X-ray scattering
SEC: Size-exclusion chromatography
SPR: Surface plasmon resonance
TA: Transcriptional activation domain
TF: Transcription factor
TFP: Trifluoperazine
TYH: Two-yeast hybrid
